# Case Report: Glucose transporter 1 deficiency syndrome misdiagnosed as bacterial meningitis

**DOI:** 10.3389/fped.2025.1698163

**Published:** 2026-01-15

**Authors:** Man Wang, Cuijin Wang, Li Shang, Yamei Feng, Dandan Huang, Qin Li, Jiwen Wang, Hui Guo, Yingyan Wang

**Affiliations:** 1 Epilepsy Center, Shanghai Deji Hospital, Qingdao University, Shanghai, China; 2 Department of Neurology, Shanghai Children’s Medical Center, Shanghai Jiao Tong University School of Medicine, Shanghai, China

**Keywords:** bacterial meningitis, glucose transporter 1 deficiency syndrome, infant, ketogenic diet, *SLC2A1*

## Abstract

**Background:**

Glucose transporter 1 deficiency syndrome (Glut1DS), caused by *SLC2A1* gene variants, is a rare neurological disorder with diverse clinical features and is highly susceptible to misdiagnosis or missed diagnosis. This article described two infant cases of Glut1DS misdiagnosed as bacterial meningitis due to atypical presentations, emphasizing the importance of early recognition.

**Method:**

Retrospective analysis of two patients with Glut1DS admitted to our hospital between July 2023 and July 2025. Clinical features, cerebrospinal fluid (CSF) profiles, genetic testing, and responses to ketogenic diet (KD) were evaluated. Both cases were preliminarily diagnosed with bacterial meningitis in local hospitals based on low CSF glucose levels.

**Results:**

Case 1: A 15-day-old male infant with fever and lethargy had persistently low CSF glucose (1.2–1.64 mmol/L; CSF/blood glucose ratio: 0.22–0.32). He was diagnosed as atypical bacterial meningitis and underwent empirical antibiotic therapy lasting over 30 days. Genetic testing confirmed *SLC2A1* variant and KD greatly improved neurodevelopment. Case 2: A 4-month-old infant with fever and recurrent seizures showed persistent CSF hypoglycorrhachia (1.1–1.37 mmol/L; CSF/blood glucose ratio: 0.19–0.36). Following unsuccessful empirical antibiotic therapy, genetic analysis revealed a pathogenic variant in *SLC2A1*. Seizure resolution and EEG improvement were achieved after KD therapy.

**Conclusion:**

low CSF glucose is a critical diagnostic clue for Glut1DS, not exclusive to CNS infections. In infants with seizures, developmental delays, or motor dysfunction, CSF analysis and targeted *SLC2A1* testing are essential. Early KD initiation upon clinical suspicion may significantly improve outcomes and prevents neurological deterioration.

## Introduction

Glucose transporter 1 deficiency syndrome (Glut1DS) is a rare inherited neurological disorder initially described by De Vivo et al. in 1991 (1). It is caused by pathogenic variants in the *SLC2A1* gene, which lead to reduced expression or dysfunction of glucose transporter protein 1 (Glut1) located on the endothelial cells of blood-brain barrier and astrocytes, reducing glucose transport into the central nervous system (CNS). This results in hypoglycorrhachia in cerebrospinal fluid (CSF) and cerebral energy deficiency, manifesting as a spectrum of neurological symptoms. In Europe and North America, the prevalence of Glut1DS is estimated at 1.65 to 4.13 cases per 100,000 live births (2, 3). The disease exhibits heterogeneous clinical phenotypes with variable severity, typically characterized by infant-onset epileptic seizures, paroxysmal movement disorders, and psychomotor developmental delay (4). The clinical presentations of Glut1DS exhibit a dynamical process, with characteristic manifestations typically emerging in early infancy, including paroxysmal ocular and head movement abnormalities accompanied by epileptic seizures. Subsequently, developmental impairments become progressively apparent. As the disease progresses, patients may develop both typical and atypical movement disorders, along with ataxia. predominant clinical features in adolescent and adult patients (5, 6).

Ketogenic diet (KD) has been well-established as the first-line therapy for Glut1DS and pyruvate dehydrogenase deficiency, with contraindications primarily in fatty acid transport and oxidation defects (7). In Glut1DS, KD effectively controls epileptic seizures and improving motor and cognitive impairments, although the response varies among individuals (8, 9). Early start of KD is strongly recommended. However, due to the rarity and complex clinical manifestations of the disease, misdiagnosis or delayed diagnosis still continue to occur, resulting in irreversible neurological damage. To raise clinical awareness and emphasize the importance of timely recognition, we retrospectively reviewed two pediatric Glut1DS cases treated with KD therapy in our hospital between July 2023 and July 2025. Both patients were initially diagnosed as bacterial meningitis in local medical centers. This study was approved by The Medical Ethics Committee of Shanghai Deji Hospital (Approval No: SDJH-LL-2024-003).

## Case presentation

### Case 1

A 15-day-old male infant was admitted to a local hospital on October 7, 2024, because of a 5-hour history of fever, lethargy, and reduced oral intake. The infant exhibited decreased arousal response and feeding volume. No cough, rhinorrhea, vomiting, choking, abnormal limb movements, or seizure were reported. He was born at full-term out of an uneventful pregnancy and delivery as the ﬁrst child of healthy parents. The family history was unremarkable.

The weight and head circumference were 3.8 kg and 35 cm respectively. The general physical examination revealed no abnormalities, except for scattered eczematous dermatitis across the body. Bilateral pupils were equal in size and shape with brisk pupillary light reflexes. Muscle tone was mildly decreased in all extremities, with normal knee and Achilles reflexes (++). No pathological reflexes or meningeal signs were observed. Initial laboratory findings included leukocytosis (WBC 17.0 × 10⁹/L), elevated C-reactive protein (CRP, 41.4 mg/L), and pyuria. CSF analysis showed RBC 6,230 × 10⁶/L, WBC 45 × 10⁶/L, protein 809.0 mg/L, and glucose 1.37 mmol/L. Blood glucose was 4.9 mmol/L, yielding a CSF-to-blood glucose ratio of 0.28 (Table 1). CSF cultures, smears, and metagenomic next-generation sequencing (mNGS) were negative for pathogens.

**Table 1:** CSF and blood glucose results in case 1.

Date of LP	Count of WBC 0–15 *10^6/L	CSF Protein 120–600 mg/L	CSF Glucose 2.2–3.9 mmol/L	Blood Glucose 3.5–5.5 mmol/L	Ratios of CSF/blood Glucose	LP Remark
2024.10.07	45	809	1.37	4.9	0.28	Traumatic
2024.10.10	9	781	1.20	4.9	0.24	
2024.10.17	7	743	1.52	4.7	0.32	
2024.10.23	10	783	1.53	5.1	0.30	
2024.11.01	5	719	1.64	6.1	0.27	
2024.11.03	10	771	1.57	5.4	0.29	
2024.11.11	8	763	1.33	6.0	0.22	

LP, lumber puncture.

The infant was initially diagnosed as bacterial meningitis and treated with meropenem combined with penicillin. After one day of treatment, his fever resolved and mental status improved. On October 10, 2024 (three days after antimicrobial therapy), blood test showed normalization of white blood cell count and CRP. CSF analysis showed normal WBC counts, elevated protein level (781 mg/L), and low glucose level (1.2 mmol/L); concurrent blood glucose was 4.9 mmol/L, yielding a CSF-to-blood glucose ratio of 0.24. Subsequent CSF analyses during hospitalization consistently showed normal cell counts, with glucose levels ranging from 1.20 to 1.57 mmol/L and CSF-to-blood glucose ratios between 0.22 and 0.32 (Table 1). Given the persistent hypoglycorrhachia despite clinical improvement, genetic testing was performed. The patient completed a four-week course of antibiotics and was discharged in stable condition.

Whole exome and mitochondrial genome sequencing revealed a heterozygous variant in the *SLC2A1* gene (NM_006516.4, c.661-663del, p.Glu221del), confirmed as a *de novo* variant by family analysis and classified as likely pathogenic (PS2 + PM4 + PM2_Supporting) according to ACMG guidelines. Combining the clinical findings of mildly decreased muscle tone and low glucose level in CSF, a diagnosis of Glut1DS was established. After excluding contraindications, the patient started classic KD therapy (2:1) in our hospital at the age of two months, with a total caloric intake of 500 kcal/day. Then, a 2:1 KD was taken and blood ketone levels were maintained around 3.0 mmol/L. Regular follow-ups were conducted, with the last telephone follow-up at the age of nine months. No significant KD-related side effects were observed. Currently, the patient's gross motor and hand function have progressed almost within normal ranges. Language development was in the preverbal phase, and his emotional and social skills were normal according to age.

### Case 2

A 4-month-old full-term male infant was admitted to a local hospital on October 1, 2023, due to a 2-day history of fever (peak temperature of 38.5 °C) and recurrent seizures for 1 day. The seizures were generalized tonic-clonic seizures: loss of consciousness, fixed gaze, cyanotic lips, clenched fists, generalized rigidity and distal tremor, lasting approximately 30 s every time and recurring up to ten times daily. The baby was born to healthy parents with an uneventful perinatal course and unremarkable family history. No special neurological symptoms were observed during fasting periods.

On admission, the infant weighed 6.5 kg with a head circumference of 41.5 cm. Physical examination revealed pharyngeal congestion. Neurological evaluation showed delayed head control and generalized hypotonia, with no other focal deficits. CSF analysis showed a slightly reddish appearance, WBC count of 76 × 10⁶/L, protein 1,897 mg/L, and glucose 1.34 mmol/L; CSF Gram stain and culture were negative (Table 2). Meanwhile, blood tests (including CRP), urinalysis, brain MRI, and echocardiography yielded no significant abnormalities. Empirical antibiotic therapy was initiated, and oral levetiracetam was started for seizure control.

**Table 2:** CSF and blood glucose results in case 2.

Date of LP	Count of WBC 0–15 *10^6/L	CSF Protein 120–600 mg/L	CSF Glucose 2.2–3.9 mmol/L	Blood Glucose 3.5–5.5 mmol/L	Ratios of CSF/blood Glucose	LP Remark
2023.10.01	76	1,897	1.34	/	/	Traumatic
2023.10.04	9	436	1.10	5.7	0.19	
2023.10.13	2	415	1.24	4.7	0.26	
2023.10.20	2	453	1.23	4.7	0.26	
2023.10.27	5	401	1.37	4.8	0.29	
2023.11.03	4	389	1.40	3.9	0.36	

LP, lumber puncture;/: No result.

Despite three days of treatment, recurrent seizures persisted, and the child was transferred to a tertiary care center. A repeat CSF analysis showed: white blood cell count 9 × 10⁶/L, protein 436 mg/L, glucose <1.1 mmol/L (Table 2); CSF Gram stain, culture and mNGS were all negative. Video electroencephalography (EEG) study revealed interictal bilateral 1.5–3 Hz medium- to high- amplitude delta activity across all channels. Scattered medium- to high- amplitude sharp wave discharges were noted in multiple channels bilaterally, most prominent in the Rolandic regions. An ictal EEG during tonic-clonic seizure demonstrated a short generalized sharp-wave discharge, followed by diffuse slow wave activity. Then, the patient continued on antimicrobial therapy with meropenem combined with linezolid, and the dose of levetiracetam was gradually escalated to control seizure. Serial CSF analyses revealed normal white cell counts, persistently low glucose levels (1.1–1.4 mmol/L), and CSF-to-blood glucose ratios ranging from 0.19 to 0.36 (as shown in Table 2). Despite these interventions, the patient continued to experience recurrent seizures at a frequency of 1–2 times daily and gene test was performed after three weeks of antibiotic treatment.

Whole-exome sequencing (WES) revealed a heterozygous variant in the *SLC2A1* gene (NM_006516, exon 3: c.136C > T, p.Gln46Ter), confirmed as a *de novo* variant by family analysis and classified as pathogenic (PVS1 + PM2_Suppoting + PS2) according to ACMG guideline. Then, the patient initiated a 2:1 modified Atkins diet (MAD) on November 20, 2024 in our center, with a maintained ratio of 1–2:1. Seizures ceased within 5 days of KD initiation. Levetiracetam was discontinued after 2 months, and EEG normalization occurred at 8 months. Developmental milestones were as follows: sitting independently at 6 months, crawling at 10 months, and independent walking at 17 months but gait instability. At the last follow-up (2 years of age), the patient demonstrated reduplicated syllables such as “dada” and “mama”.

## Discussion

Glut1DS is characterized by three core clinical symptoms: epilepsy, paroxysmal movement disorders, and developmental delays in motor and cognitive function (8). According to the consensus guidelines published by the International Glut1DS Study Group in 2020 (5), the diagnosis relies on the following three criteria: First, clinical manifestations, including seizures, paroxysmal or persistent movement disorders (e.g., ataxia, dystonia, choreoathetosis) triggered by fasting, fatigue, or physical exertion, and delays in motor and intellectual development; Second, CSF biochemical abnormalities, defined as CSF glucose concentration < 2.8 mmol/L and CSF-to-blood glucose ratio < 0.6; Third, genetic evidence, demonstrated by identification of a pathogenic *SLC2A1* variant. Diagnostic criteria require fulfillment of at least one of the following: All three criteria (clinical features+CSF abnormalities+*SLC2A1* variant); Clinical features and genetic evidence; In cases where *SLC2A1* genetic testing is not performed or results are negative, a clinical diagnosis may be established if criteria 1 and 2 are met and exclusion criteria (e.g., mitochondrial disorders, hypoglycemia, or other metabolic diseases) are satisfied. Due to the highly heterogeneous manifestations and individual variability in clinical phenotypes, the diagnosis of Glut1DS is often late. Therefore, it is necessary to improve the early recognition and diagnostic capabilities among clinicians.

Normal blood glucose level with concomitant reduced CSF glucose concentration is a key metabolic hallmark of Glut1DS. To ensure reliable measurements, lumbar puncture should be performed after four- to six-hour fast, preferably before breakfast, with concurrent assessment of blood glucose levels immediately prior to lumbar puncture (10). Studies have shown that CSF glucose levels in patients with Glut1DS typically range from 0.9 to2.9 mmol/L (16.2 to 52.0 mg/dL), consistently below 3.3 mmol/L (60 mg/dL), with CSF-to-blood glucose ratios ranging from 0.19 to 0.59 (11). Most patients exhibit CSF glucose levels below 2.2 mmol/L (40 mg/dL) and CSF-to- blood glucose ratio below 0.4. Regarding diagnostic value, the absolute reduction in CSF glucose concentration is more reliable than CSF-to-blood glucose ratio, and CSF glucose level correlates with clinical disease severity (12). In this case series, one patient exhibited fluctuating absolute CSF glucose levels from 1.2 to 1.64 mmol/L, with CSF to blood glucose ratios ranging from 0.22 to 0.32 (Table 1). The other patient demonstrated CSF glucose levels of 1.1 to 1.37 mmol/L and CSF to blood glucose ratios from 0.19 to 0.36 (Table 2). Both cases displayed typical CSF glucose profile of Glut1DS, defined by marked CSF hypoglycorrhachia with preserved systemic glucose homeostasis. In clinical practice, children presenting with unexplained epilepsy, epilepsy with psychomotor delay, or movement disorders, should undergo standardized lumbar puncture at the earliest opportunity to enhance the early recognition of Glut1DS. In children with persistently low CSF glucose levels, particularly below 2.2 mmol/L, clinicians should heighten clinical suspicion of Glut1DS.

Bacterial meningitis is a common pediatric neurological condition, with CSF analysis serving as the primary diagnostic basis (13). Typical CSF findings include elevated white blood cell count (predominantly neutrophils), elevated protein, and reduced glucose or CSF-to-serum glucose ratio (14). However, CSF glucose levels can vary widely, from normal to markedly low, depending on pathogen, disease stage, and prior administration of intravenous glucose-containing fluids (15). Early in the course or after antibiotic exposure, CSF profiles may be atypical. Importantly, hypoglycorrhachia is not specific to bacterial meningitis; it also occurs in CNS inflammation, neoplasms, hypoglycemia, ischemia, hypoxia, and Glut1DS (16). Thus, in cases with high clinical suspicion, integrated interpretation of clinical features and laboratory data is essential for accurate diagnosis. In our report, Patient 1 had elevated blood infection markers, whereas Patient 2 showed no significant inflammatory parameters. The initial CSF pleocytosis in both was likely due to traumatic lumbar puncture, as white blood cell counts normalized within 3 days of treatment. Nevertheless, both infants were initially diagnosed with bacterial meningitis and received empirical antibiotics, likely due to: (1) acute fever with neurological symptoms, lethargy in Patient 1 and seizures in Patient 2, could be typical clinical presentations of CNS infections; (2) initial CSF showing elevated white cells, elevated protein, low glucose, and neutrophil predominance, was consistent with bacterial meningitis despite possible traumatic contamination; (3) the phenomenon that CSF leukocytosis may be absent in neonates or young infants with bacterial meningitis (17); (4) concurrent infections, such as respiratory and urinary tract infections, may occur in Glut1DS individuals, and fever can provoke seizures and complicate the initial diagnostic suspicion; (5) the rarity of fasting-induced Glut1DS symptoms in regularly fed infants, masking its typical presentations when fasting.

In patients exhibiting atypical clinical manifestations of CNS infection following antibiotic therapy and persistent CSF hypoglycorrhachia, traumatic lumbar puncture must be rigorously excluded and findings interpreted alongside clinical data to avoid diagnostic misinterpretation. Comprehensive evaluation of clinical parameters is critical for accurate differential diagnosis. All patients with confirmed or highly suspected bacterial meningitis should receive a full course of antibiotics, while clear criteria for discontinuation remain undefined, with current guidance largely empirical (18). Notably, some children continue to exhibit abnormal CSF profiles despite completing standard antibiotic regimens (19, 20). In the present cases, persistent hypoglycorrhachia prompted extended antibiotic therapy. Combined integration of clinical features, laboratory results, and treatment response necessitated timely diagnostic reevaluation and therapeutic adjustment. Inadequate recognition of clinical manifestations and laboratory profiles of Glut1DS might lead to misdiagnoses, missed diagnoses, and inappropriate management. Therefore, it is essential to strengthen educational initiatives and training on rare neurological disorders like Glut1DS in primary hospitals.

Genetic testing is crucial for confirming the diagnosis of Glut1DS, especially for individuals with mild or atypical findings. The *SLC2A1* gene, typically inherited autosomal dominant, occurs predominantly *de novo* variants (21). Missense, deletions, insertions, splice site, and nonsense harbor pathogenic variants accounting for approximately 84% cases. Single- or multi-exon and whole-gene deletions account for ∼13%, and rare intronic or start-codon variants have also been reported (22–24). Therefore, for patients with a strong clinical suspicion but negative results on conventional exome sequencing and copy number variant screening, multiplex ligation-dependent probe amplification (MLPA) analysis and genetic investigation of non-coding regions should be considered (24). Generally, genetic variant types correlate with phenotypic severity: missense variants typically result in mild to moderate phenotypes; splice variants, nonsense variants, insertions, deletions, and exon deletions tend to cause moderate to severe manifestations; and complete gene deletion is associated with the most severe phenotype (25, 26). Notably, approximately 5%–15% of patients globally present with typical Glut1DS phenotypes and CSF glucose reduction despite negative *SLC2A1* testing results (8). This discrepancy may stem from variants in non-coding regions or alterations affecting downstream processes-such as Glut1 transcription, translation, or protein activation-meaning the absence of detectable pathogenic *SLC2A1* variants does not definitively exclude Glut1DS. In our study, both patients exhibited *de novo* *SLC2A1* variants in a sporadic setting: the first carried an in-frame deletion and presented with mild hypotonia and CSF hypoglycorrhachia, while the second, exhibiting recurrent seizures, had a pathogenic nonsense variant. Genetic testing is essential for confirming Glut1DS, as it not only identifies causative *SLC2A1* variants but also enables prediction of disease severity based on variant type. Although genotype-phenotype correlations show heterogeneity and a minority of patients with classic Glut1DS lack identifiable pathogenic variants (8), genetic analysis remains indispensable for definitive diagnosis.

KD is an effective therapy for Glut1DS, providing the developing brain with an alternative metabolic fuel. Early initiation of KD after diagnosis is closely associated with improved clinical outcomes (27, 28). KD may achieve seizure cessation or reduction, withdrawal of antiseizure medications, and normalization of abnormal EEG findings in majority of patients (29). It may also improve neurodevelopmental manifestations, including cognitive and language impairments as well as motor dysfunction, although responses are variable (11). In children under two years, the classical KD is preferred to sustain higher ketone levels and meet the brain's energy demands. For adolescents, adults, or those with poor adherence to strict regimens, MAD, is a viable alternative. Provided it is well tolerated, long-term KD is recommended and can be maintained into adulthood (5). The latest consensus recommendations from International Ketogenic Diet Study Group suggests that regular blood monitoring should be conducted to maintain serum *β*-hydroxybutyrate levels within the target range of 2∼5 mmol/L (30). In our cohort, Patient 1 initiated KD at 2 months of age, with *β*-hydroxybutyrate stabilized near 3.0 mmol/L; by 9 months, gross motor, fine motor, and cognitive development were nearly normal. Patient 2 began KD immediately after diagnosis: seizures ceased after 5 days, antiseizure medications were discontinued after 2 months, and EEG normalized by 8 months, with no subsequent seizures, although mild motor delay persisted. These cases further underscore the importance of early diagnosis and timely intervention in improving prognosis. Notably, neither patient experienced severe adverse events during KD therapy, demonstrating excellent tolerability.

In summary, decreased CSF glucose levels are not unique to CNS infections. When infants present with unexplained epilepsy, epilepsy with psychomotor delay, or movement disorders, prompt lumbar puncture should be performed to facilitate diagnosis. Gene testing is critical for definitive diagnosis, particularly in clinically suspected cases. Continuous persistent CSF-to-blood glucose ratio below 0.33 warrants high clinical suspicion for Glut1DS. As a treatable neurogenetic disorder, early recognition and diagnosis of Glut1DS are important. KD is the first-line treatment, and its initiation should be prioritized once Glut1DS is suspected to alleviate symptoms and improve clinical outcomes. Systematic education and training should be implemented to improve physician awareness of rare neurogenetic disorders including Glut1DS, thereby preventing missed and delayed diagnoses.

## Data Availability

The original contributions presented in the study are included in the article/Supplementary Material, further inquiries can be directed to the corresponding authors.
